# Early results from implementation of HIV pre-exposure prophylaxis (PrEP) with long-acting injectable cabotegravir for people with barriers to oral strategies, Italy, December 2024 to August 2025

**DOI:** 10.2807/1560-7917.ES.2025.30.39.2500739

**Published:** 2025-10-02

**Authors:** Davide Moschese, Rozenn Esvan, Chiara Fusetti, Virginia Barchi, Maria Vittoria Cossu, Alessandro Giacinta, Samuel Lazzarin, Giulia Terracini, Francesco Caruso, Giulia Micheli, Donatella Maddalena, Giulia Del Duca, Francesco Petri, Gianluca Frate, Maddalena Matone, Damiano Farinacci, Andrea Giacomelli, Paolo Faccendini, Debora Visigalli, Sara Passacantilli, Agostino Riva, Andrea Antinori, Andrea Gori, Valentina Mazzotta

**Affiliations:** 1 Department of Infectious Diseases, Luigi Sacco University Hospital, ASST Fatebenefratelli Sacco, Milan, Italy; 2 Regional AIDS Reference Center, National Institute for Infectious Diseases, Lazzaro Spallanzani IRCCS, Rome, Italy; 3 Department of Biomedical and Clinical Sciences, Università degli Studi di Milano, Milan, Italy; 4 Hospital Pharmacy Unit, National Institute for Infectious Diseases, Lazzaro Spallanzani IRCCS, Rome, Italy; 5 Pharmacy Unit, Luigi Sacco University Hospital, ASST Fatebenefratelli Sacco, Milan, Italy; 6 Health Direction, National Institute for Infectious Diseases, Lazzaro Spallanzani IRCCS, Rome, Italy; 7 Centre for Multidisciplinary Research in Health Science (MACH), Milan, Italy

**Keywords:** pre-exposure prophylaxis, HIV, cabotegravir, PrEP, implementation, real-world evidence

## Abstract

Between December 2024 and August 2025, two Italian HIV/STI clinics offered long-acting injectable cabotegravir (CAB-LA) for HIV prevention in 265 individuals, representing the first European real-world implementation. Participants were prioritised based on vulnerabilities compromising oral pre-exposure prophylaxis (PrEP), such as poor adherence, comorbidities and behavioural barriers. Adherence to injections exceeded 95%, tolerability was favourable and only 1.5% discontinued for drug-related reasons. These findings demonstrate the feasibility of CAB-LA in European clinical services and support its use in specific populations.

Despite remarkable advances in HIV prevention, more than 100,000 new diagnoses continue to be reported annually in the World Health Organization (WHO) European Region [1]. Between December 2024 and August 2025, two Italian centres specialising in HIV/sexually transmitted infection (STI) care offered long-acting injectable cabotegravir (CAB-LA) for pre-exposure prophylaxis (PrEP) in 265 individuals. CAB-LA was recently recommended in European guidelines (2024), but no real-world European data outside clinical trials have been reported to date. The programme specifically prioritised people with vulnerabilities that could compromise the effectiveness of oral PrEP, including suboptimal adherence, relevant comorbidities, and structural or behavioural barriers. In this report, we present the characteristics of enrolled participants, their reasons for accessing CAB-LA, and early outcomes related to adherence, tolerability, safety and treatment discontinuations.

## Implementation approach

**Figure 1:**
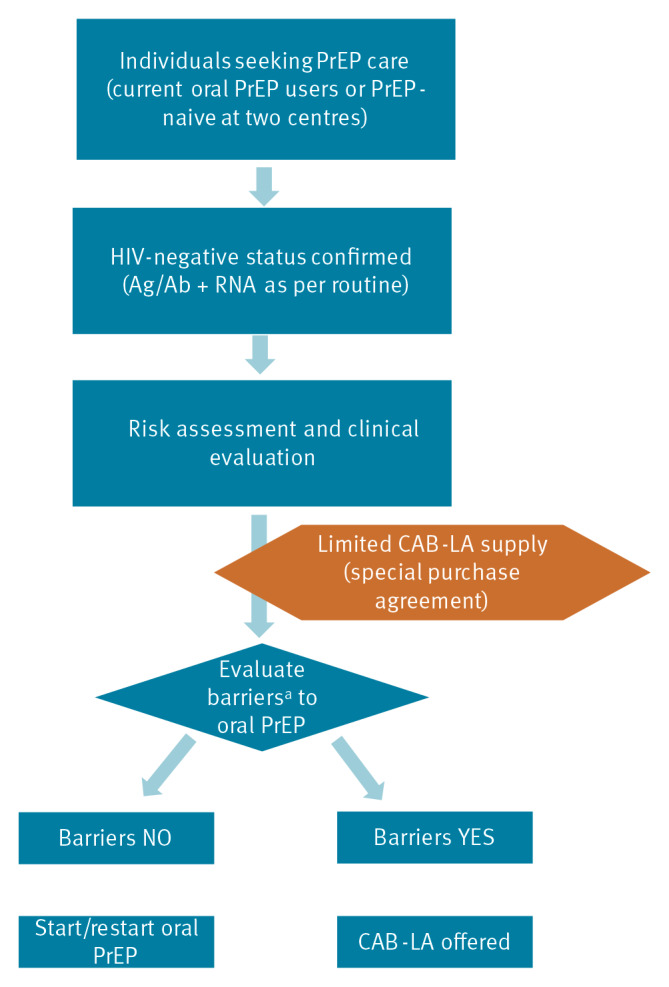
Flow chart of the selection of study participants to receive long-acting injectable cabotegravir, Italy, December 2024–August 2025 (n = 265)

Because the pilot program provided a limited number of vials, the two centres (National Institute for Infectious Diseases Lazzaro Spallanzani, Rome, and Luigi Sacco University Hospital, Milan) developed an operational protocol to prioritise candidates (Figure 1). CAB-LA was offered only to PrEP users (current or new) with documented barriers to oral PrEP: suboptimal oral PrEP adherence, PrEP tolerability issues, renal impairment, osteopenia/osteoporosis, psychiatric illness, chemsex practices, involvement in sex work, malabsorption/dysphagia, pill burden or drug–drug interactions, allergy, transgender/cisgender female identity, and refusal or discontinuation of oral PrEP. Participants were identified by clinicians in two large centres, provided informed consent, and received CAB-LA according to European clinical guidelines [2,3].

Ag/Ab: antigen/antibodies; CAB-LA: cabotegravir long-acting; PrEP: pre-exposure prophylaxis.

^a^ Barriers include adherence, tolerability, toxicity, DDI, behavioural/structural barriers and refusal/discontinuation.

CAB-LA (600 mg) was administered as an initial injection followed by a second dose after 4 weeks, and then every 8 weeks (± 7 days). No oral lead-in was administered. Delayed or missed injections were managed through guideline-based re-initiation or oral PrEP bridging. At each injection visit, clinicians systematically recorded injection-site pain (using a numeric rating scale, grade 0–3) and the presence of nodules and fever. Any other systemic adverse events, e.g., headache, gastrointestinal intolerance, insomnia, reduced libido, were captured if reported spontaneously. HIV Ag/Ab and HIV-RNA tests were performed at each injection.

## Population characteristics

Between December 2024 and August 2025, 265 individuals initiated CAB-LA for PrEP in Milan and Rome. Most were cisgender men (92.8%; n = 246) identifying as gay, bisexual and other men-who-have-sex-with-men (GBMSM), with a median age of 38 years (interquartile range (IQR): 33–47). Nearly all (89.4%; n = 237) had previous oral PrEP experience. The socio-demographic and behavioural characteristics of the individuals, as well as reasons to access CAB-LA, are shown in the Table.

**Table:** Characteristics of the study population receiving long-acting injectable cabotegravir, Italy, December 2024–August 2025 (n = 265)

Population	n	%
**Gender identity**		
Cisgender men	246	92.8
Cisgender women	10	3.8
Transgender women	9	3.4
**Sexual orientation**		
GBMSM	247	93.2
Heterosexual	15	5.7
Bisexual women	3	1.1
**Country of origin**		
Italy	237	89.4
Abroad	28	10.6
**Education**		
University	189	71.3
High school	67	25.3
Middle school	9	3.4
**Working condition**		
Employed	241	90.9
Student	11	4.2
Retired	7	2.6
Unemployed	6	2.3
**Sex work** (self-reported)		
Yes	10	3.8
No	255	96.2
**Sexual partners** (last 3 months)		
< 5	44	16.6
6–10	118	44.5
11–30	83	31.3
> 30	15	5.7
Missing data	5	1.8
**BMI**		
< 18.5	3	1.1
18.5–24.9	157	59.2
25–29.9	91	34.3
≥ 30	14	5.3
**Circumcision**		
Uncircumcised	238	89.8
Circumcised	15	57.7
Not applicable	12	4.5
**History of STIs**		
Yes	161	60.8
No	104	39.2
**PrEP history **		
Already on oral PrEP	237	89.4
PrEP-naive/discontinued	28	10.6
**Reasons for accessing CAB-LA for PrEP**^a^		
Suboptimal adherence	114	43.0
Chemsex use	37	14.0
Discontinuation/refusal of oral PrEP	29	10.9
Tolerability issues	28	10.6
Renal impairment	24	9.1
Psychiatric illness	24	9.1
TGW/CGW identity	19	7.2
Osteopenia/osteoporosis	13	4.9
Malabsorption/dysphagia	8	3.0
Sex worker	10	3.8
Pill burden/DDIs	3	1.1
Allergy	2	0.8
**Discontinuations** (n = 15)		
Engage in monogamous relationship	6	2.3
Injection site pain	2	0.8
CNS (insomnia or reduced libido)^b^	2	0.8
Relocation outside the city	2	0.8
GI intolerance^b^	1	0.4
Weight gain^b^	1	0.4
Unknown	3	1.3

BMI: body mass index; CAB-LA: cabotegravir long-acting; CNS: central nervous system; DDIs: drug-drug interactions; GBMSM; gay, bisexual and other men who have sex with men; GI: gastrointestinal; PrEP: pre-exposure prophylaxis; STIs: sexually transmitted infections; TGW/CGW: transgender women/cisgender women.

^a^ Reasons for eligibility may concomitantly be present in the same participant.

^b^ Causes may concomitantly be present in the 15 participants who discontinued.

## Discontinuation of treatment

**Figure 2:**
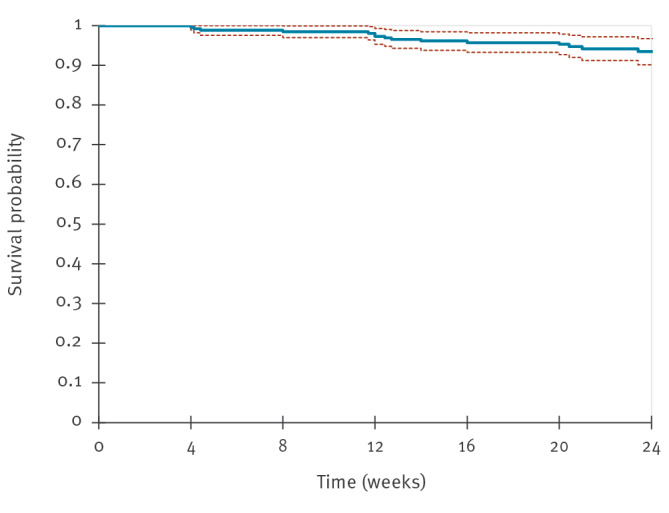
Kaplan-Meier curve for the probability of participants remaining on long-acting injectable cabotegravir, Italy, December 2024–August 2025 (n = 265)

The median follow-up was 22.7 weeks (IQR: 20.0–28.3). Overall, 15 participants (5.7%) discontinued long-acting PrEP: six for engagement in a monogamous relationship (2.3%); four (1.5%) for drug-related reasons (two due to injection-site pain, one due to gastrointestinal intolerance and insomnia, one due to weight gain and libido reduction), two (0.8%) moving out of the city and three (1.3%) were lost to follow-up. The 24-week probability of remaining on CAB-LA was 93% (95% confidence interval (95% CI): 90–97%) (Figure 2).

The blue solid line represents the Kaplan-Meier survival estimate; the red dashed lines indicate the 95% confidence interval, showing that the probability of remaining on CAB-LA stayed consistently high (93% at week 24).

## Breakthrough infections

No HIV breakthrough infections were observed. No discordant results between the HIV Ag/Ab test and the HIV viral load were detected.

## Adherence

**Figure 3:**
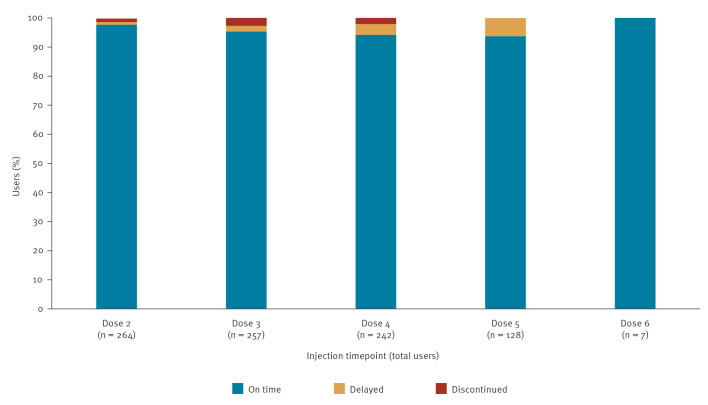
Adherence to PrEP injection schedule and discontinuations of participants on long-acting injectable cabotegravir, Italy, December 2024–August 2025 (n = 265)

Adherence to the injection schedule was consistently high. At the second injection, 97.7% (258/264) were on time, with 95.3% (245/257) and 94.2% (228/242) remaining within the window at the third and fourth doses. Across follow-up, 16 participants experienced minor delays (within 65 days) considered clinically tolerable, while seven with longer delays (within 77 days) were managed by sexual abstinence (n = 6) or oral PrEP with tenofovir disoproxil fumarate/emtricitabine (TDF/FTC) bridging (n = 1), restarted with two tablets the first day and the one daily until the date of the next injection (Figure 3).

Proportions of participants receiving each scheduled injection within the recommended window (28 ± 7 days for dose 2; 56 ± 7 days for doses 3–4), showing consistently high adherence.

## Adverse effects

**Figure 4:**
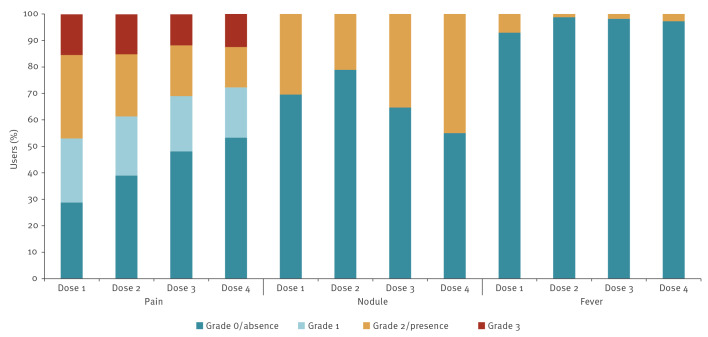
Local adverse reactions to cabotegravir injections among participants, Italy, December 2024–August 2025 (n = 265)

Injection site pain was the most common local reaction. The proportion of individuals reporting no pain increased from 28.7% (76/265) at the first dose to 53.3% (129/242) at the fourth, while the proportion of individuals experiencing moderate-to-severe pain (grades 2–3) declined accordingly. Nodules were reported in 21–45% of participants across doses, but they were consistently mild to moderate in severity and did not lead to treatment discontinuation. Fever was uncommon (≤ 7%; 18/265), mainly occurring after the first injection (Figure 4).

Distribution of pain grades (0–3) at doses 1–4, showing decreasing frequency and intensity over time. Nodules were reported in 21–45% of participants, with no clear trend, and fever was uncommon (≤ 7%), typically occurring after the first dose.

Only a limited number of patients had reached the sixth (n = 7) dose at the time of analysis.

Body weight remained stable through week 20. Among 165 participants with longitudinal paired data, the median change was 0.0 kg (IQR: −1.0 to + 1.0), with only 7 individuals (4.2%) experiencing shifts greater than ± 5 kg (three gained + 6, + 9, and + 11 kg, and four lost –7, –9, and –10 kg in two cases). Consistently, BMI remained stable across weight categories, with negligible mean changes from baseline to week 20 in underweight (–0.04), normal weight (–0.02), overweight (–0.05) and obese (–0.03) participants.

Central nervous system events were rare: one case of post-injection headache was considered tolerable, while insomnia and reduced libido each contributed to different treatment discontinuations.

## Discussion

Evidence from clinical trials has shown the superior efficacy of CAB-LA over daily oral PrEP [4,5], but implementation data from European healthcare systems have so far been lacking. This programme represents the first European real-world implementation of CAB-LA, demonstrating that CAB-LA can be delivered in routine practice with high levels of feasibility and acceptability, particularly among individuals with vulnerabilities that compromise oral PrEP. By prioritising those with suboptimal adherence, comorbidities such as renal impairment or osteoporosis, and behavioural factors including chemsex or sex work, this programme addressed populations unlikely to achieve adequate protection with oral strategies [6].

Previous concerns have centred on whether adherence to repeated injections could be maintained outside trial conditions [7,8]. In our cohort, adherence to the injection schedule remained consistently high across follow-up, with 98% and 96% on time at the third and fourth doses, respectively. Deviations were infrequent and generally short, while longer delays were successfully managed by sexual abstinence or oral PrEP bridging, ensuring sustained protection. These data provide reassurance that the long-acting formulation can maintain adherence in real-world settings, overcoming a key limitation of oral PrEP.

Tolerability has been another major consideration for injectable PrEP, particularly due to injection-site reactions [9]. In our programme, pain was frequent at initiation but declined with subsequent doses, was generally mild to moderate, and rarely led to discontinuation. Fever occurred in the minority of participants and was without clinical consequence, while nodules were frequently reported but were mild to moderate in severity and did not result in treatment discontinuation. Together, these findings confirm that CAB-LA is well tolerated in practice and that injection-site events are unlikely to be a barrier to scale-up. Our tolerability findings align with clinical trials HPTN 083, 084 and 077 [4,5,10], where injection-site pain, nodules and occasional fever were the most frequent adverse effects recorded, largely mild/moderate and rarely causing discontinuation.

Metabolic safety of CAB-LA, including potential weight changes, remains under investigation [11,12]. Our analysis showed stable weight through week 20, with only a small minority experiencing changes greater than 5 kg, distributed in both directions and without a systematic signal. Central nervous system events were very rare, with only insomnia and reduced libido contributing to discontinuation. These results strengthen the safety profile of CAB-LA, complementing data from trials with real-world evidence.

Finally, retention in PrEP programmes is critical, as discontinuations may reduce effectiveness at the population level [13,14]. In our cohort, 15 participants (5.7%) discontinued CAB-LA during follow-up, but only five (1.9%) for drug-related reasons. The majority were unrelated to treatment or due to loss to follow-up, highlighting the need for supportive services to maximise persistence. Importantly, no HIV breakthrough infections occurred, underscoring the preventive effectiveness of CAB-LA when delivered in real-world conditions. Moreover, the absence of discordant results between the HIV Ag/Ab test and viraemia supports the 2024 International Antiviral Society (IAS) recommendations on monitoring CAB-LA users with HIV Ag/Ab testing alone [15].

This report describes an early real-life experience from two referral centres with limited CAB-LA supply, where participants were prioritised due to barriers to oral PrEP; therefore, generalisation of the findings may be limited. In addition, the small sample size prevented powered analysis, and follow-up was short; systemic adverse events were mainly captured if reported spontaneously.

## Conclusions

Taken together, our findings provide the first European demonstration that CAB-LA can be successfully integrated into HIV prevention services. By addressing individuals with barriers to oral PrEP, this pilot experience highlights the potential of CAB-LA to close persistent gaps in protection and informs ongoing scale-up efforts across European Union/European Economic Area countries.

## Data Availability

Data are available upon reasonable request to the corresponding author.
